# Development of male sterile lines of CMS chilies (*Capsicum annuum* L.) from F_1_ hybrids

**DOI:** 10.1270/jsbbs.22042

**Published:** 2023-05-17

**Authors:** Maneechat Nikornpun, Kridsada Sukwiwat, Kittisak Wongsing, Jutamas Kumchai

**Affiliations:** 1 Department of Plant and Soil Sciences, Faculty of Agriculture, Chiang Mai University, Chiang Mai 50200, Thailand; 2 Department of Agricultural Extension, Ministry of Agriculture and Cooperatives, Thailand

**Keywords:** cytoplasmic male sterility, general combining ability, maintainer, specific combining ability

## Abstract

Selfing and crossing methods were used to develop the cytoplasmic male sterility (CMS) lines from 2 elite F_1_ hybrids of CMS hot chilies. The pungency of the CMS lines was improved by backcrossing with the B cultivar. The first and second backcrossed progenies of the CMS lines showed significantly higher capsaicin contents than the F_1_ hybrids. One good female line K16 × BBC2 (K16), was selected and backcrossed with 3 good maintainer cultivars, C5, C9 and C0. Some incomplete male sterility of pollens was demonstrated in the F_1_ hybrids and the 1^st^ backcrossed progenies while the partial sterility disappeared by the stage of the second and third generations of backcrossing. When K16 and P32 were crossed with restorers, fruit yields and yield components of certain F_1_ hybrids, parental lines and commercial varieties were significantly different. Heterosis of yield and yield components of the F_1_ hybrid chilies was significant. When K16 was used as a female parent, positive and significant heterosis of the F_1_ hybrids was the same as P32. Moreover, significant GCA of the restorer lines, C7, C8 and C9, was observed in some horticultural characteristics. Furthermore, significant differences of the specific combining ability of some characteristics were observed in a few F_1_ hybrids.

## Introduction

Cytoplasmic male-sterility (CMS) in chilies has previously been documented (Budar and Pelletier 2001, Mankare and Korekar 2017, Peterson 1958, Schnable and Wise 1998, Shifriss 1997, Vesselina *et al.* 2010, Woong 1990). The CMS lines—female CMS lines, maintainer lines and restorer lines, have been found to be precious genetic materials and as such are not available to chili breeders either by requesting or through purchasing. The CMS lines were provided worldwide by the World Vegetable Center, AVRDC, after the donations from some Korean companies. The lines were made available in Thailand as of 2006 but the development of the lines by the authors at Chiang Mai University had started back in 2000. Subsequently, commercial F_1_ hybrids were the only source for CMS lines. CMS is a maternally inherited trait encoded by the mitochondrial genome, and CMS disturbs functional pollen production, a trait that has been used for hybrid F_1_ seed production. Molecular biology studies revealed that CMS is associated with newly-made chimeric mitochondrial open reading frames (ORFs), which interfere with mitochondrial function and pollen development (Kim *et al.* 2001, Lin *et al.* 2013). CMS is associated with two different ORFs, *orf507* and *ψatp6-2*. These ORFs potentially impair ATP synthesis activity in the cytoplasm, resulting in non-functional pollen. The conventional process for hybrid development is tedious and time consuming and involves three seasons: developing testcross F_1_ (in the first season), growing and obtaining F_2_ seeds (in the second season) and examining segregation in the F_2_ progenies (in the third season) (Yeh *et al.* 2016). CMS markers and *Rf* locus associated markers were developed to distinguish N-cytoplasm from S-cytoplasm (Ji *et al.* 2014, Jindal *et al.* 2020, Jo *et al.* 2010, Kim and Kim 2005, Lin *et al.* 2015, Liu *et al.* 2013, Yeh *et al.* 2016). This has been found to be useful in the screening of the CMS individuals in the early seedling stage, thus facilitating economic production of hybrid seeds. The World Vegetable Center, developed male sterile lines and maintainers of CMS chili seeds and distributed them worldwide. The use of CMS lines lowers the cost of hybrid seed production by at least 40% when compared with manual emasculation and pollination (Lin *et al.* 2013). Cytoplasmic male sterility is commonly used in the F_1_ hybrid seed production of chilies (Yang *et al.* 2008). Searching for usable fertility restorers and stable maintainers are major tasks but these lines are needed for efficient and low-cost seed production. Nowadays, several seed companies use the genetic mechanism—*rfrf* on a large scale for producing chili hybrids (*Capsicum annuum* L.), whereas, the cytoplasmic source is used mainly for breeding pungent (S*Rfrf*) chili hybrids (Shifriss 1997). Chiang Mai University has identified CMS lines of chilies and distributed them to seed companies. Our new cultivars using CMS and *Rf* are sold in China. Chiang Mai University has distributed the chili germplasm to 10 private sector organizations and Horticultural Research Institute at the Yunnan Academy of Agricultural Science, North Suburban, Kunming, Yunnan, China, and material transfer agreements were signed between 2009 and 2018. The Horticultural Research Institute at the Yunnan Academy of Agricultural Science has successfully produced and registered two outstanding F_1_ hybrid chilies commercially. In addition the seeds of one variety, CT117, are available for commercial markets.

Sprague and Tatum (1942) defined the general combining ability (GCA) as the average performance of a genotype in a series of hybrid combinations. Parents showing a high average combining ability in crosses are considered to have a good GCA while if their potential to combine well is bounded to a particular cross, they are considered as having good specific combining ability (SCA). Moreover, they defined SCA as those cases in which certain hybrid combinations perform better or poorer than would be expected on the basis of the average performance of the parental inbred lines. Griffing (1956) defined SCA as an indication of loci with dominance variance (non-additive effects) and all the three types of epistatic interaction components if epistasis were present. They include additive × additive, additive × dominance and dominance × dominance interactions. From a statistical point of view, the GCA is the main effect and the SCA is an interaction effect. The GCA correlates with the activity of genes which are largely additive in their effects as well as additive × additive interactions (Kulembeka *et al.* 2012). The expression of heterosis is highly associated with the SCA of crosses. The study of combining ability is useful in predicting the performance of hybrids. Combining ability analysis also identifies elite parents which are general combiners over a series of cross combinations and good specific combiners to exploit heterosis (Nsabiyera *et al.* 2013). Heterosis is a genetic phenomenon resulting from heterozygosity (Kuroda *et al.* 1998). Mid-parent heterosis is the difference between the hybrid and the mean of the two parents and this is often expressed as a percentage of the mid-parent performance (Falconer and Mackay 1996, Lamkey and Edwards 1999).

The objectives of this study were to develop male sterile and maintainer lines of CMS chilies (*Capsicum annuum* L.) from F_1_ hybrids. The F_1_ hybrids from the developed CMS lines and restorers would be tested for their general combining abilities, specific combining abilities and heterosis in relation to yield and yield components.

## Materials and Methods

A series of experiments for improvement of cytoplasmic male sterile (CMS) and maintainer lines of chilies (*C. annuum* L.) were carried out at Chiang Mai University, Chiang Mai, Thailand in 2007–2011. The soil type of the area was sandy loam, and the temperature during 2007–2011 had maximum temperatures between 37.1–41°C and minimum temperatures between 10.9–13.4°C. The data on rain fall/year during 2007–2011 was in the range of 1,070.2–1,449.5 mm. Field management practices for all experiments were as follows, 30 day-old seedlings were transplanted into the field, which included 2 tons of cow manure, in addition to 31.25 kg/ha, of fertilizer (15N-15P-15K) mixed into the soil. Liquid fertilizer which contained 300 g of 15N-15P-15K, 130 g of 13N-0P-46K and 150 g of 0N-52P-34K, 5 g of trace elements Mg 9.0%, Fe 4.0%, Mn 4.0%, Cu 1.5%, Co 0.05%, Zn 1.5%, B 0.5% and Mo 0.1%, mixed into 200 liters of water was also used. Insecticides; imidacloprid, fipronil sulfur and methomyl were used at recommended rates once a week.

The fertility status of the chilies was visually observed at the flowering stage for the presence or absence of anthers and checked for pollen production and fertility. A fertility scoring method was used to evaluate the fertility of the pollen (Gulyas *et al.* 2006, Pakozdi *et al.* 2002, Rai *et al.* 2001, Yoon *et al.* 2006). Thirty plants were grown for each F_1_ hybrid in the same place and under the same cultural practices as mentioned above. The seedlings were randomly planted with 30 replications. Pollen from open flowers was stained with 1% acetocarmine to score for pollen fertility. Pollen viability was rated based on the red stained color and the morphology of the pollen. Capsaicin levels of the chili fruits was analyzed by Anan’s method (Anan *et al.* 1996).

### Selection and improvement of capsaicin levels of CMS lines by backcrossing

#### Development of CMS lines

**Fig. 1.**
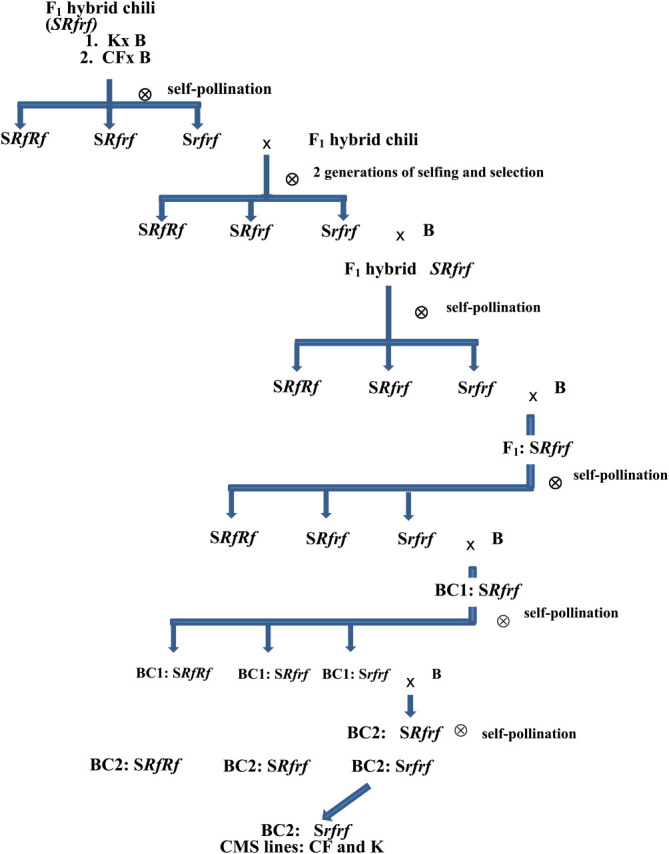
Development of CMS lines of chilies (C. *annuum*) from elite hybrids (K & CF—imported female CMS, S*rfrf*, B–a pure line, hot Thai Chili, BC1–1^st^ backcross, BC2–2^nd^ backcross, S–sterile cytoplasm, *Rf*–restorer gene, *rf*–recessive sterile gene).

Cytoplasmic male sterile-hot chilies (*C. annuum* L.) were developed from elite hybrids (Fig. 1), which are high-yielding but do not have high pungency (Milerue and Nikornpun 2000). They were produced from CMS lines, K and CF, and crossed with a local cultivar, B. Subsequently, they were segregated for sterility upon selfing. The two F_1_ hybrids, K × B and CF × B were heterozygous (*Rfrf*) for restorer genes and, were selfed before being selected for CMS plants for three generations. The horticultural characteristics of the selected CMS progenies (S*rfrf*) were not good for Thai chilies. Therefore, the CMS plants were crossed with the B cultivar (N*RfRf*), local hot chilies (Nikornpun 2011). The progenies were then selfed again. They were then backcrossed with the B cultivar for two generations. The CMS lines, CF and K (BC2:S*rfrf*) were also then selected. K, B, CF, K14, K16, C5, C9, C0 and P32 are abbreviation of the following varieties—K (an imported female CMS, S*rfrf*), B (a pure line, hot Thai chili), CF (an imported female CMS, S*rfrf*), K14 (the backcrossed female CMS, S*rfrf*), K16 (the backcrossed female CMS, S*rfrf*), C5 (a maintainer, N*rfrf*), C9 (a maintainer, N*rfrf*), C0 (a maintainer, N*rfrf*) and P32 (a standard female CMS, S*rfrf*).

#### Improvement of CMS lines for yield and capsaicin levels

In 2002, the capsaicin contents of K and CF were improved by using the hot chili, B cultivar in a backcrossing method (Nikornpun 2011). The CMS progenies from the first and second backcrosses were tested for capsaicin content in comparison with the F_1_ hybrid generation in a randomized complete block design with 3 replications. The seedlings were planted in double row beds, 50 × 50 cm with a plot size of 4.5 m^2^. Desirable CMS plants were selected from the second backcrossed progenies. Three male sterile lines, CF × BBC_2_, K14 × BBC_2_ and K16 × BBC_2_ were selected as the most desirable.

One good female line K16 × BBC_2_ (K16) was selected and crossed with three good maintainer cultivars, C5, C9 and C0. The progenies were backcrossed with respective parents for three generations. The CMS lines were released to private seed companies and the Horticultural Research Institute at the Yunnan Academy of Agricultural Science, North Suburban, Kunming, Yunnan, China.

### Testing of stabilities of chili maintainers for the CMS K16

**Fig. 2.**
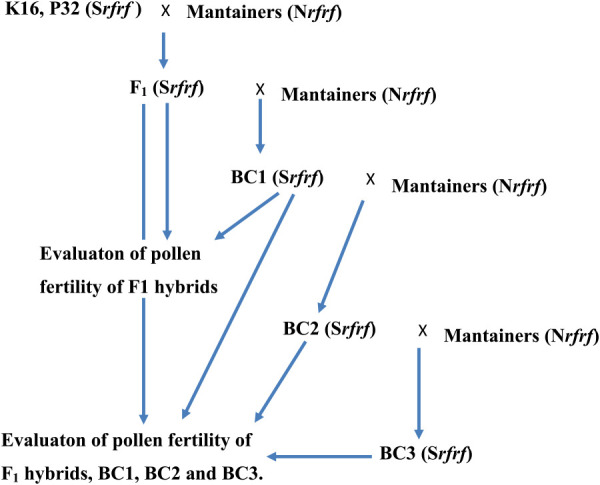
Evaluation of stabilities of chili maintainers for the CMS lines K16 and P32 (K16–the backcrossed female CMS, S*rfrf*, P32–a standard female CMS, S*rfrf*, BC1–1^st^ backcross, BC2–2^nd^ backcross, BC3–3^rd^ backcross, S–sterile cytoplasm, N–normal cytoplasm, *Rf*–restorer gene, *rf*–recessive sterile gene).

Stabilities of chili maintainers (N*rfrf*) for the CMS line K16 (S*rfrf*)—were tested in 2009–2010 (Fig. 2). Two experiments were carried out. In the first experiment, the CMS line K16 was crossed with the maintainers (Nikornpun 2011). The F_1_ hybrids—(S*rfrf*)—were backcrossed with the maintainers, C5, C9, and C0. Ten F_1_ hybrids were further obtained. The F_1_ hybrids from K16-8 were crossed with C0-4 and those from K16-10 were crossed with C9-5. Thirty plants of the F_1_ hybrids and the first backcrossed progenies (BC1, S*rfrf*) were also randomly grown for pollen fertility scoring.

In the second experiment, the line, P32 (S*rfrf*) (Seungchon CMS/6 * Saegochu/5 * PBC 385) which is a male sterile line from the World Vegetable Center, was used as a standard female line (Wongsing 2015) (Fig. 2). K16 and P32 were crossed with the maintainers, C5-2 and C0-7. The F_1_ hybrids were backcrossed with the maintainers for two generations. The F_1_ hybrids and the three backcrossed progenies (BC1, BC2 and BC3) were evaluated for pollen fertility.

Four F_1_ hybrids, K16 × C5-2, P32 × C5-2, K16 × C0-7 and P32 × C0-7, were obtained. They were backcrossed with chili maintainers, C5-2 and C0-7. The first backcrossed progenies, K16 BC_1_ × C5-2, K16BC_1_ × C0-7, P32BC_1_ × C5-2 and P32BC_1_ × C0-7, were also obtained. The BC_1_ progenies were backcrossed with the respective maintainers. The second backcrossed progenies, K16BC_2_ × C5-2, K16BC_2_ × C0-7, P32BC_2_ × C5-2 and P32BC_2_ × C0-7, were then obtained. Next the BC_2_ progenies were backcrossed with their respective maintainers. The third backcrossed progenies, K16BC_3_ × C5-2, K16BC_3_ × C0-7, P32BC_3_ × C5-2 and P32BC_3_ × C0-7, were also obtained. Thirty plants of the F_1_ hybrids, and the first, second and third backcrossed progenies were randomly grown for pollen fertility scoring.

### Heterosis and combining abilities of yield and yield components of CMS lines, restorer lines and F_1_ hybrids of chilies by using the CMS K16 and P32

The third backcrossed progenies of CMS lines, K16C5, K16C0, P32C5 and P32C0, were crossed with the fertility restorer lines, C7, C8 and C9. Twelve F_1_ hybrids; K16C5 × C7, K16C5 × C8, K16C5 × C9, K16C0 × C7, K16C0 × C8, K16C0 × C9, P32C5 × C7, P32C5 × C8, P32C5 × C9, P32C0 × C7, P32C0 × C8 and P32C0 × C9, were obtained (Wongsing 2015). The F_1_ hybrid seeds were grown with the parental lines and commercial varieties in a randomized complete block design with three replications. The seedlings were planted in double row beds, 50 × 50 cm with a plot size of 4.5 m^2^. The horticultural characteristics were evaluated at harvesting time. The general combining abilities and specific combining abilities for the horticultural characteristics were calculated (Kempthorne 1973). Moreover, an analysis of the combining ability was carried out by using R software, while the heterosis for the horticultural characteristics were also calculated (Chen *et al.* 2003).

## Results

### Selection and improvement of capsaicin levels of CMS lines by backcrossing

#### Development of CMS lines

**Fig. 3.**
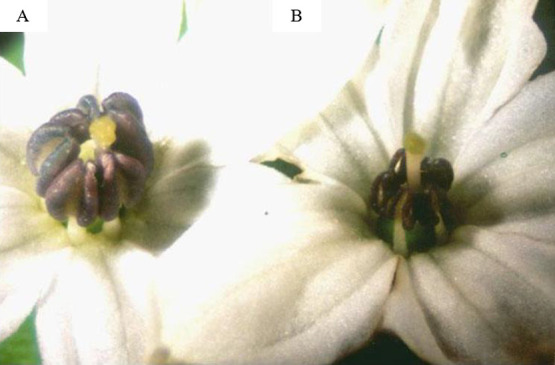
Fertile flowers (A) and sterile flowers (B) of chilies.

Two elite F_1_ hybrids of CMS hot chilies; K × B and CF × B were used for the development of cytoplasmic male sterility lines. They were selfed and selected for male sterile lines. A pollen fertility scoring method was used to identify prospective male sterile genotypes. The male sterile lines were backcrossed with B cultivar. They were selfed again and the CMS lines, CF and K were selected from the selfed progenies. The flowers of the CMS lines had small anthers when compared with fertile anthers (Fig. 3).

### The improvement of CMS lines for capsaicin levels

**Fig. 4.**
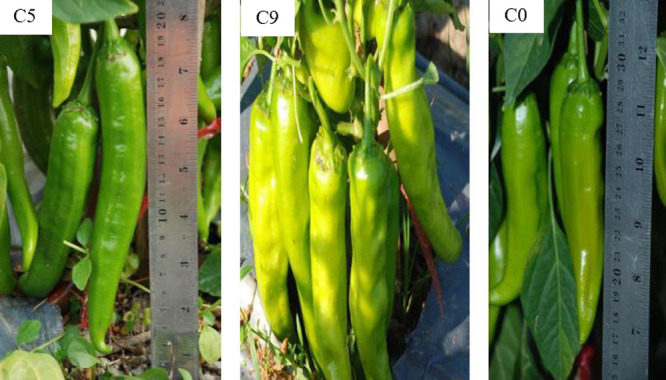
Fruit morphology of maintainer lines, C5, C9 and C0.

The local hot chili, B cultivar was used to improve the fruit capsaicin of the selected CMS lines, CF, K14 and K16, by backcrossing (Nikornpun 2011). The fruit capsaicin of the hybrids and the backcrossed progenies of both hybrids showed significant variation (Table 1). The values ranged from 1,313–10,110 scoville units. The highest capsaicin levels among the hybrids and the backcrossed progenies were observed in K16 × BBC_2_ and CF × BBC_2_ (11,110 and 9,147 scoville units, respectively). These two backcrossed progenies had capsaicin levels significantly higher than the hybrids. Most of the capsaicin levels of the second backcrossed progenies were significantly higher than the first backcrossed progenies. The pungency of the CMS lines was improved by backcrossing with the B cultivar. One good female line K16 × BBC_2_ was selected. It was crossed with three good maintainer cultivars, C5, C9 and C0. The fruit morphologies of the maintainer cultivars are shown in Fig. 4. Superior breeding lines were released to private seed companies in Thailand and the Horticultural Research Institute at the Yunnan Academy of Agricultural Science, North Suburban, Kunming, Yunnan, China in 2010.

### Testing of stabilities of chili maintainers for the CMS K16

The stabilities of chili maintainers for the CMS line K16 were tested in 2009–2010 by backcrossing (Nikornpun 2011, Wongsing 2015). The F_1_ hybrids and the first, second and third backcrossed progenies were evaluated for their male sterility levels by using pollen viability. In 2009, the F_1_ hybrids and the 1^st^ backcrossed progenies, all possessed nonviable pollen with some contamination of fertile pollens (Table 2). Prospective male parents had recessive maintainer genes in the nucleus (*rfrf*), and normal cytoplasm (N).

In 2010, K16 and P32 were crossed and backcrossed for three generations with chili maintainers (Wongsing 2015). The CMS P32, was used as a standard check variety. The F_1_ hybrids and the first backcrossed progenies in which K16 was a female parent, demonstrated nonviable pollen with some contamination of fertile pollens (Table 2). The contamination of the incomplete male sterile plants disappeared in the second and third backcrossed progenies. However, when P32 was used as the female parent, the F_1_ hybrids, and the first to the third backcrossed progenies demonstrated nonviable pollen without contamination.

The maintainers were stable but some incomplete male sterility was seen in the F_1_ hybrids and the first backcrossed progenies when K16 was used as the female parent. This problem disappeared with more generations of backcrossing.

### Heterosis and combining abilities of yield and yield components of maintainers, restorers and F_1_ hybrids using the CMS lines, K16 and P32

#### Fruit yield and yield components

**Fig. 5.**
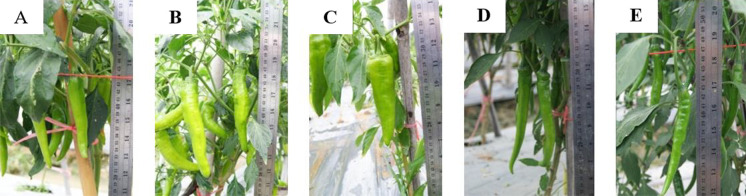
Fruit morphology of high yielding F_1_ hybrids chili (K16C5 × C8 (A), K16C0 × C8 (B), P32C5 × C8 (C)) and commercial varieties (D and E) [Varieties; K16 (the backcrossed female CMS, S*rfrf*), C8 (a maintainer, N*rfrf*), and P32 (a standard female CMS, S*rfrf*) CJ, CM (commercial cultivars)].

The maintainers, C5 and C0, which were used to develop the CMS lines K and P32, were tested for heterosis and combining abilities (Table 3). This was needed to confirm that the backcrossed female CMS with the maintainers would express the heterosis and good combining abilities in the F_1_ hybrids. Chili fruit yield and yield components of the F_1_ hybrids, parental lines and commercial varieties were significantly different (Table 3). The yields ranged from 16 to 61 t/ha. The F_1_ hybrid K16C5 × C8 gave the highest yield, which was significantly higher than most of the F_1_ hybrids, male and female parents and one commercial variety. However, it was not significantly different from some other F_1_ hybrids. The second highest yielding varieties, K16C0 × C8 and P32C5 × C8 gave 50 and 48 t/ha, respectively. The F_1_ hybrids were cayenne chilies (*Capsicum annuum* L.) with long green fruits (Fig. 5). They yielded significantly more than some F_1_ hybrids, female parents and some male parents. However, they were not significantly different from the commercial varieties. The number of fruit per plant and fruit weight per plant of the highest yielding F_1_ hybrid K16C5 × C8, were significantly higher than most of the F_1_ hybrids, male and female parents and one of the commercial varieties (Table 3). It was not significantly different from some other F_1_ hybrids and one of the commercial varieties. However, the average weight of fruit, fruit length and the fruit width of this hybrid were not significantly different from most of the F_1_ hybrids, female parents and a few of or most of male parents. The average weight of fruit was significantly higher than one of the commercial varieties. The fruit width of the F_1_ hybrid, K16C5 × C8, was significantly higher than the commercial varieties, while not significantly different from the commercial varieties for fruit length.

#### Heterosis

The heterosis for yield and yield components of the F_1_ hybrids were significantly different (Table 3). The highest yielding variety, K16C5 × C8, and the second highest yielding variety, K16C0 × C8, demonstrated positive and significant differences of heterosis in yield and yield components, number of fruit per plant, fruit weight per plant, average weight of fruit, fruit length and fruit width. When line K16 was used as a female parent, positive and significant heterosis was found in 17 F_1_ hybrids for yield and yield components. When line P32 was used as a female parent, positive and significant heterosis was found in 16 F_1_ hybrids for yield and yield components. The female line K16 was as good as the standard female line P32.

#### General combining ability and specific combining ability

The restorer line C7, showed significant difference in GCA for the number of fruit per plant. C8 showed significant differences in GCA for yield, number of fruit per plant and fruit weight per plant. Additionally, C9 demonstrated significant differences in GCA for fruit length (Table 3).

Significant differences in specific combining ability (SCA) were observed in a few F_1_ hybrids (Table 3). The F_1_ hybrid—K16C5 × C8 demonstrated significantly higher SCA for four characteristics—yield, number of fruit per plant, average weight of fruit and fruit weight per plant. The F_1_ hybrid—K16C0 × C9 demonstrated significantly higher SCA for four characteristics—yield, average weight of fruit, fruit weight per plant and fruit length. The F_1_ hybrid—P32C5 × C7 demonstrated significantly higher SCA for three characteristics—yield, average weight of fruit and fruit length. The F_1_ hybrid—P32C0 × C9 demonstrated significantly higher SCA for three characteristics—number of fruit per plant, fruit weight per plant and average weight of fruit. The F_1_ hybrid—K16C0 × C7 demonstrated significantly higher SCA for one characteristic—number of fruit per plant. The F_1_ hybrid, P32C0 × C7 demonstrated significantly higher SCA for two characteristics—fruit weight per plant and fruit length.

## Discussion

Cytoplasmic male sterility is a maternally inherited trait in which a plant fails to produce functional anthers, pollen grains, or male gametes. The CMS system responds to nuclear genes, *Rf* and *rf*, that interact with N or S cytoplasm (Kim *et al.* 2001, 2007, Peterson 1958, Vesselina *et al.* 2010). A single gene controls the CMS expression in some cases while many genes control male sterility in other cases (Gulyas *et al.* 2006, Wang *et al.* 2004). There would be no differences of CMS trait and *Rf* locus in this work from those in previous studies. However, the CMS trait and *Rf* locus in this work are among the combinations of genes that comprise the average Thai chilies. We have hereby proven that extraction of S cytoplasm, *Rf* and *rf* from the hybrids was possible. We also found that the segregation of progenies of the CMS lines followed Mendel’s laws of inheritance, indicating a single *Rf* gene. Moreover, the development of line K16 by conventional breeding was a long and tedious process. Three CMS lines, CF × B BC_2_10, K14 × B BC_2_ and K16 × B BC_2_ (K16), were developed from two elite F_1_ hybrids of CMS hot chilies. The pungency of the male sterile lines was improved by backcrossing with the B cultivar. K16 progenies were selected and then backcrossed with good maintainers, C5, C9 and C0. In addition, the stability of C5 and C0, was high in the second and third generations of backcrossing. Furthermore, the expression of CMS traits might require a certain environment. The homozygous maintainers (N*rfrf*) were used for the backcrossing. As the backcrossing was progressing, the incomplete CMS trait appeared only in the F_1_ hybrids and the 1^st^ backcrossed progenies, while it disappeared in the second and third generations of backcrossing. Additionally, the homozygosity of genes in advanced backcrosses might influence the expression of the CMS traits (this is only our own observations, there is no supporting empirical documentary evidence). There are different extant reports on the control of CMS traits. A single gene controls the CMS expression in some cases, while many genes control male sterility in other cases (Gulyas *et al.* 2006, Wang *et al.* 2004). The line K16 could be used as a good female line in terms of the stability of maintainers. When line K16 was used as a female parent, positive and significant heterosis of the F_1_ hybrids was more or less the same as the standard P32 line. The CMS line K16 was as good as the P32 for the development of F_1_ hybrids. The female line K16, developed here was as good as P32 in terms of yields and yield components. The development involved the generation of large seedling populations for the selection of the best individuals. Modern breeding technologies have used DNA markers to identify genes responsible for CMS in chili (Ji *et al.* 2014, Jindal *et al.* 2020, Jo *et al.* 2010, Kim and Kim 2005, Kim *et al.* 2006, Kumar *et al.* 2007, Yeh *et al.* 2016). However, we were not able to find appropriate DNA markers for our chilies. When K16C0, K16C5, P32C0 and P32C5 were crossed with restorers, the highest yielding variety (K16C5 × C8) and the second highest yielding variety (K16C0 × C8), demonstrated positive and significant heterosis in yield and yield components. Heterosis is the basis for improvement of crop yield which is due to superior gene content of inbred parents and, contributes to superior performance (Mather 1955). Significant heterosis and heterobeltiosis for yield and horticultural characteristics of hybrid chilies have also been reported more generally (Abrham *et al.* 2017, Ganefianti and Fahrurrozi 2018, Kumar *et al.* 2014, Nagaraju *et al.* 2017, Patel *et al.* 2010, 2014, Prasath and Ponnuswami 2008, Rekha *et al.* 2016, Shrestha *et al.* 2011). The testing of maintainers is needed to confirm that the backcrossed female CMS lines with the maintainers would accurately express the heterosis in the F_1_ hybrids.

Significant differences in SCA were observed in a few F_1_ hybrids. The estimation of the SCA of parents is an important indicator of their potential for generating superior F_1_ hybrids. GCA and SCA determine the efficacy of breeding for improvements in given traits and can be used to identify lines to be used as parents in a breeding program (Rekha *et al.* 2016). The restorer lines, C7, C8 and C9 showed significant differences in GCA for some characteristics.

## Author Contribution Statement

Conceptualization, M.N.; methodology, M.N., K.S., K.W. and J.K.; validation, M.N.; visualization, M.N.; formal analysis, K.S., K.W., W.K. and J.K.; writing-original draft preparation, M.N.; writing-review and editing, M.N., V.G. All authors have read and agreed to the published version of the manuscript.

## Figures and Tables

**Table 1.** Capsaicin contents of fruit of F_1_ hybrids, the first and second backcrossed chilies

Variety	Capsaicin/g of fruit (scoville unit)	Heterosis (%)
F_1_ hybrid		
CF × B^1^	7,493^d2^	–
K × B	1,313^f^	–
First backcrossed (BC_1_)		
CF × BBC_1_^3^	8,063^c^	7.60
K14 × BBC_1_	5,290^e^	302.89
K16 × BBC_1_	4,703^e^	258.18
Second backcrossed (BC_2_)		
CF × BBC_2_	9,147^b^	13.44
K14 × BBC_2_	7,220^d^	36.48
K16 × BBC_2_	10,110^a^	114.96
CV (%)	16.97	–

^1^ Varieties; K (an imported female CMS, S*rfrf*), B (a pure line, hot Thai chili), CF (an imported female CMS, S*rfrf*), K14, K16 (the backcrossed female CMS, S*rfrf*), ^2^ Means followed by the same letters indicate no differences at P ≤ 0.05 by the least significant difference, ^3^ BC_1_–1^st^ backcross, BC_2_–2^nd^ backcross.

**Table 2.** Fertility of F_1_ hybrids and the backcrossed progenies of chilies

Variety	Number of plants						Contamination^1^ (%)			Genotypes of male parents
	Fertile			Sterile						
	2009	2010		2009	2010		2009	2010		
F_1_ hybrid										
K16^2^-1 × C5-1	1	–		22S + 7I^3^	–		24.14	–		N*rfrf*^4^
K16-2 × C5-1	0	–		25S + 5I	–		16.67	–		N*rfrf*
K16-3 × C5-2	0	–		30S	–		0	–		N*rfrf*
K16-4 × C5-2	0	–		24S + 6I	–		20	–		N*rfrf*
K16-5 × C5-2	0	–		24S + 6I	–		20	–		N*rfrf*
K16-6 × C9-1	6	–		18S + 6I	–		25	–		N*rfrf*
K16-7 × C9-2	1	–		22S + 7I	–		24.14	–		N*rfrf*
K16-8 × C9-2	1	–		22S + 7I	–		24.14	–		N*rfrf*
K16-9 × C0-1	0	–		30S	–		0	–		N*rfrf*
K16-7 × C0-2	0	–		26S + 4I	–		13.33	–		N*rfrf*
K16 × C5-2	–	0		–	22S + 8I		–	26.67		N*rfrf*
P32 × C5-2	–	0		–	30S		–	0		N*rfrf*
K16 × C0-7	–	0		–	20S + 10I		–	33.33		N*rfrf*
P32 × C0-7	–	0		–	30S		–	0		N*rfrf*
Average contamination							16.7	15		
First backcrossed progenies										
K16^2^-8 BC1XC0-4	0			26S + 4I			13.33			N*rfrf*
K16-10BC1XC9-5	0			30S			0			N*rfrf*
K16 BC1XC5-2		0			30S			0		N*rfrf*
K16BC1XC0-7		0			25S + 5I			16.67		N*rfrf*
P32BC1XC5-2		0			30S			0		N*rfrf*
P32BC1XC0-7		0			30S			0		N*rfrf*
Average contamination							6.67	4.14		
Second backcrossed progenies										
K16BC2XC5-2		0			30S			0		N*rfrf*
K16BC2XC0-7		0			30S			0		N*rfrf*
P32BC2XC5-2		0			30S			0		N*rfrf*
P32BC2XC0-7		0			30S			0		N*rfrf*
Average contamination								0		
Third backcrossed progenies										
K16BC3XC5-2		0			30S			0		N*rfrf*
K16BC3XC0-7		0			30S			0		N*rfrf*
P32BC3XC5-2		0			30S			0		N*rfrf*
P32BC3XC0-7		0			30S			0		N*rfrf*
Average contamination								0		

^1^ Contamination = % of incomplete male sterile plants, ^2^ Varieties; K16 (the backcrossed female CMS, S*rfrf*), C0, C5, C9 (maintainers, N*rfrf*), and P32 (a standard female CMS, S*rfrf*). ^3^ 22S + 7I = 22 male sterile plants + 7 incomplete male sterile plants, ^4^ N*rfrf* = normal cytoplasm with homozygous recessive CMS genes.

**Table 3.** Heterosis and combining ability of yield and horticultural characteristics of chilies in 2010

Variety	Yield					No fruit/pt			
	t/ha^1^	GCA^2^	SCA^3^	%H^4^		No/pt^5^	GCA	SCA	%H
F_1_ hybrids									
K16^7^C0 × C7	33 c–g^8^	–	–815.467	–4.24		55 c	–	1.861**	75.53**
K16C0 × C8	50 ab	–	120.853	128.80**		70 b	–	–0.806	106.90**
K16C0 × C9	26 e–h	–	694.614*	–16.88		31 gh	–	–1.056*	2.17
K16C5 × C7	36 b–f	–	–486.933	15.08**		41 ef	–	–2.694**	40.11**
K16C5 × C8	61 a	–	1,632.107**	218.08**		66 b	–	4.639**	107.29**
K16C5 × C9	16 h	–	–1145.175**	–44.55		21 i	–	–1.944**	–26.01**
P32C0 × C7	40 b–e	–	569.066	15.28		51 cd	–	0.861	63.83**
P32C0 × C8	39 b–f	–	–1409.174**	76.40**		64 b	–	–4.472**	88.18**
P32C0 × C9	25 e–h	–	840.107**	–20.28**		33 g	–	3.611**	8.70**
P32BC_3_C5 × C7	43 b–d	–	733.333*	36.87**		46 de	–	–0.028	54.80**
P32BC_3_C5 × C8	48 a–c	–	–343.787	149.89**		64 b	–	0.639	100.00**
P32BC_3_C5 × C9	20 g–h	–	–389.546	–30.53		24 hi	–	–0.611	–15.61**
Female									
C0-7	29 d–h	–	–	–		32 g	–	–	–
C5-2	23 f–h	–	–	–		29 gh	–	–	–
Male									
C7-3	40 b–e	256.96	–	–		30 gh	1.028**	–	0.856
C8-2	15 h	2,122.560**	–	–		35 fg	18.694**	–	–1.034
C9-3	33 c–g	2,379.521**	–	–		29 gh	–19.722	–	–
Commercial var.									
CJ	43 b–d	–	–	–		70 b	–	–	–
CM	50 ab	–	–	–		92 a	–	–	–
CV (%)	35.98					47.67			

Variety	Fruit wt/pt					Ave.fruit wt			
	Kg/pt^6^	GCA^2^	SCA^3^	%H^4^		g	GCA	SCA	%H
F_1_ hybrids									
K16^6^BC_3_C0 × C7	1.24 c–g^7^	–	–0.193**	–4.24**		22.52 e–g	–	–5.684	–45.93**
K16BC_3_C0 × C8	1.91 ab	–	0.029	128.80**		27.21 c–g	–	0.896	8.26*
K16BC_3_C0 × C9	0.98 e–h	–	0.164*	–16.88		32.31 b–f	–	4.787**	–16.35*
K16BC_3_C5 × C7	1.37 b–f	–	–0.115	15.08**		33.41 b–e	–	0.042	–16.88**
K16BC_3_C5 × C8	2.32 a	–	0.386**	218.08**		35.01 b–d	–	3.539*	47.85**
K16BC_3_C5 × C9	0.59 h	–	–0.271**	–44.55**		29.11 c–f	–	–3.581*	–21.7*
P32BC_3_C0 × C7	1.50 b–e	–	0.135*	15.28**		29.29 c–f	–	1.628	–29.6**
P32BC_3_C0 × C8	1.47 b–f	–	–0.334**	76.40**		23.01 d–g	–	–2.759	–8.43
P32BC_3_C0 × C9	0.94 e–h	–	0.119**	–20.28**		28.12 c–g	–	1.132	–27.21**
P32BC_3_C5 × C7	1.63 b–d	–	–0.174*	36.87**		35.62 bc	–	4.014*	–11.38
P32BC_3_C5 × C8	1.82 a–c	–	–0.081	149.89**		28.04 c–g	–	–1.676	18.39*
P32BC_3_C5 × C9	0.74 g–h	–	–0.062	–30.53		28.59 c–g	–	–2.338	–23.10**
Female									
C0-7	1.09 d–h	–	–	–		33.59 b–e	–	–	–
C5-2	0.88 f–h	–	–	–		30.69 c–f	–	–	–
Male									
C7-3	1.51 b–e	0.061	–	–		49.69 a	0.856	–	–
C8-2	0.58 h	0.502**	–	–		16.67 g	–1.034	–	–
C9-3	1.26 c–g	–0.563**	–	–		43.66 ab	0.178	–	–
Commercial var.									
CJ	1.62 b–d	–	–	–		23.11 d–g	–	–	–
CM	1.9 ab	–	–	–		20.8 fg	–	–	–
CV (%)	35.98					24.61			

Variety	Fruit length					Fruit width			
	cm	GCA^2^	SCA^3^	%H^4^		cm	GCA	SCA	%H
F_1_ hybrids									
K16^7^BC_3_C0 × C7	10.26 ef^8^	–	–2.459**	–34.51**		2.16 ab	–	–0.024	–60.95**
K16BC_3_C0 × C8	13.87 b–d	–	1.036**	3.90**		2.16 ab	–	0.083	9.35**
K16BC_3_C0 × C9	14.77 a–d	–	1.423**	–5.30**		2.12 ab	–	–0.059	–9.06**
K16BC_3_C5 × C7	13.08 cd	–	–1.043	–12.08**		2.29 ab	–	0.002	0.96**
K16BC_3_C5 × C8	15.15 a–c	–	0.911**	20.61**		2.28 ab	–	0.098	18.65**
K16BC_3_C5 × C9	14.89 a–c	–	0.132	0.51		2.18 ab	–	–0.1	–4.17**
P32BC_3_C0 × C7	15.13 a–c	–	2.717**	–3.39**		2.10 ab	–	–0.072	–9.82**
P32BC_3_C0 × C8	9.98 f	–	–2.556**	–25.27**		1.93 b–d	–	–0.132*	–2.44**
P32BC_3_C0 × C9	12.89 c–e	–	–0.161	–17.39***		2.37 a	–	0.203**	1.36**
P32BC_3_C5 × C7	15.17 a–c	–	0.785**	2.02*		2.26 ab	–	0.094	–0.22
P32BC_3_C5 × C8	15.12 a–c	–	0.609*	20.34**		2.01 a–c	–	–0.049	4.77**
P32BC_3_C5 × C9	13.63 b–d	–	–1.394**	–7.98**		2.12 ab	–	–0.044	–6.95**
Female									
C0-7	14.79 a–d					2.26 ab			
C5-2	13.21 cd					2.15 ab			
Male									
C7-3	16.53 ab	–0.251	–	–		2.39 a	0.037	–	–
C8-2	11.91 d–f	–0.132	–	–		1.69 cd	–0.069*	–	–
C9-3	16.41 ab	0.384**	–	–		2.41 a	0.032	–	–
Commercial var.									
CJ	16.91 a	–	–	–		1.57 a	–	–	–
CM	14.24 a–d	–	–	–		1.65 cd	–	–	–
CV (%)	12.94					11.04			

^1^ ton/hectare, ^2^ general combining ability, ^3^ specific combining ability, ^4^ heterosis, ^5^ number of fruit/plant, ^6^ kg/plant, ^7^ varieties; K16 (the backcrossed female CMS, S*rfrf*), C0, C5, C7, C8, C9 (maintainers, N*rfrf*), P32 (a standard female CMS, S*rfrf*), CJ, CM (commercial cultivars), ^8^ means followed by the same letters indicate no differences at P ≤ 0.05 by the least significant difference, *, ** significant difference at P ≤ 0.05 and P ≤ 0.01 levels, respectively.
